# Radial Retinotomies with Endodiathermy for Severe Proliferative Vitreoretinopathy: Short-Term Results

**DOI:** 10.1155/2016/2594574

**Published:** 2016-02-28

**Authors:** Zhaoxin Jiang, Suo Qiu, Bingsheng Lou, Miaoli Lin, Junlian Tan, Xiaofeng Lin

**Affiliations:** Zhongshan Ophthalmic Center, Sun Yat-sen University, Guangzhou 510080, China

## Abstract

*Purpose.* Retinal redetachment of silicone oil-filled eyes continues to be a frustrating condition that typically requires retinectomy. We proposed radial retinotomy as a potentially less invasive surgery. Here, we preliminarily explored its feasibility, efficacy, and safety.* Methods.* Totally 9 eyes of 9 consecutive patients were included in a prospective noncomparative trial. A series of retinotomies were created by endodiathermy in a radial pattern to relax the foreshortened retina. The eye was refilled with fresh silicone oil. The treated eyes were examined via visual acuity (VA) tests, tonometry, slit-lamp microscopy, and fundus photography during a 6-month observation period.* Results.* The procedure was completed in an average of 28 minutes from silicone oil removal to fresh silicone oil placement. Fundus photography demonstrated that 7 of the 9 eyes (78%) exhibited retinal reattachment. On average, VA was significantly improved within the first 2 weeks (*P* = 0.02) and remained stable for the following 6 months. The change in intraocular pressure was not significant (*P* = 0.76), and no adverse event was observed (0%).* Conclusion.* Radial retinotomies with endodiathermy were shown to be feasible, effective, and safe in selected cases of inferior contracted retina without vitreous base fibrosis over a 6-month observation period. This trial is registered with NCT02201706.

## 1. Introduction

Retinal redetachment, especially in a silicone oil-filled eye, continues to be among the most frustrating and challenging conditions faced by vitreoretinal surgeons. The recurrence rate of retinal detachment in silicone oil-filled eyes is approximately 20% [1–4]. The major cause of redetachment in these cases is attributed to originally severe retinal injury and subsequently inferior proliferative vitreoretinopathy (PVR); in such cases, stiffness and foreshortening of the retina are often observed [5–8].

**Figure 1:**
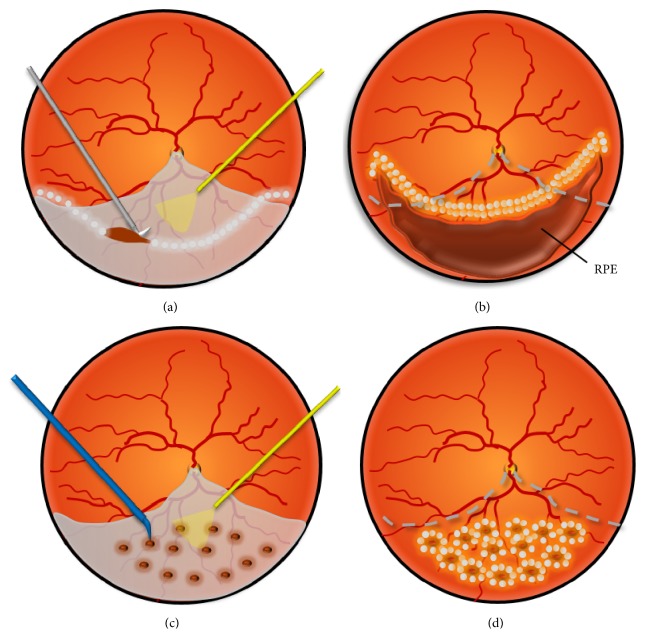
Schematic drawings of traditional relaxing retinectomy and radial retinotomies with endodiathermy. ((a), (b)) The relaxing retinectomy should extend into the normal retina on each end of the contractive area, resulting in a large area of defective retina and exposed RPE. (c) A series of retinotomies are created via endodiathermy in a radial pattern to relax the stiffened and foreshortened retina. All of the retinotomies are limited to the detached retina. (d) These individual round retinal incisions elongate in an oval fashion during the water-air exchange. In turn, this elongation enables the foreshortened retina to stretch and recover the RPE.

Conventionally, relaxing retinotomies and retinectomies, first introduced by Machemer in 1979, are often required to treat retinal shortening (Figures 1(a) and 1(b)) [9, 10]. To reverse this shortening, the retinectomy should extend into the normal retina on each end of the contractive area, resulting in the exposure of a large area of the RPE and further injury to the normal retina [11]. Potentially serious complications, such as intraoperative hemorrhage from the choroid, large retinal defects, and hypotony, are associated with these conventional procedures [12–16]. Therefore, a new surgical method that balances reattachment with preservation of the retina is greatly needed.

Here, we propose a novel procedure consisting of radial retinotomies with endodiathermy as a potentially less invasive alternative to this conventional approach (Figures 1(c) and 1(d)). Briefly, rather than a sequential cut with scissors, endodiathermy is applied to relax the shortened retina. Specifically, based on a complete vitrectomy, endodiathermy is used to perforate the centers of contraction to relax the asteroidal retina. Subsequently, a series of retinotomies are created via endodiathermy in a radial pattern to relax the stiff and foreshortened retina. All of the retinotomies are limited to the detached retina. This procedure allows the individual round retinal incisions to elongate in an oval fashion. In turn, the retina is permitted to stretch, resulting in the coverage of the RPE below the previously detached, foreshortened retina.

Theoretically, this modified method of retinotomies avoids extensive cutting of the normal retina and preserves the shortened retina to cover the underlying RPE, consequently reducing certain complications of relaxing retinectomy. To our knowledge, no reports have discussed this modified surgery. Therefore, we conducted an exploratory study to preliminarily evaluate the feasibility, efficacy, and safety of this procedure.

## 2. Methods

### 2.1. Study Design

This study was a single-center, open-label, nonrandomized, prospective clinical trial (registered with the identifier NCT02201706 at ClinicalTrials.gov) designed to preliminarily evaluate the feasibility, efficacy, and safety of a modified procedure of radial retinotomies with endodiathermy for the treatment of recurrent retinal detachment in patients with severe proliferative vitreoretinopathy.

The described research adhered to the tenets of the Declaration of Helsinki. Approval was obtained from the Sun Yat-sen University Medical Ethics Committee (Zhongshan Ophthalmic Center Medical Ethics, number 2014MEKY018). The trial was conducted from January 2014 to June 2015. Informed consent was obtained from all individual participants included in the study.

### 2.2. Patients

Patients with severe proliferative vitreoretinopathy for whom conventional treatment methods had failed were enrolled. To limit the inconsistency of ocular conditions, only patients with inferior retinal redetachment in silicone oil-filled eyes were recruited. The inferior retinal redetachment was located at 3–9 o'clock. Moreover, the primary retinal detachment was caused by ocular trauma. The frequency of silicone oil tamponade was only once. All patients were from 5 to 60 years old.

Patients were excluded if they had an unobservable fundus due to severe corneal opacity, a nonfunctional contralateral eye, only one eye, or serious heart, lung, liver, or kidney dysfunction.

### 2.3. Surgical Technique

All of the surgeries were performed by the same surgeon (XFL). The surgical procedures varied depending on the vitreoretinal pathology; however, certain general principles were followed (Figures 1(c) and 1(d)). In brief, after the silicone oil was removed through the scleral incision, complete vitrectomy and membrane peeling were performed (Alcon Laboratories, Inc., Fort Worth, TX, USA). Next, endodiathermy was performed to perforate the centers of contraction to relieve focal contractions. Subsequently, endodiathermy was applied continually to generate a series of retinotomies in a radial or fan-shaped pattern to completely mobilize the stiffened retina. Afterwards, the retina regained elasticity and floated in the vitreous body. During the water-air exchange, the individual circular incisions were elongated into an oval shape to cover the RPE below the previously detached, foreshortened retina. Finally, three rows of argon blue-green endophotocoagulation were applied to the edges of the retinectomy, and silicone oil was injected to completely fill the vitreous cavity.

### 2.4. Outcome Measures

The primary outcome was complete retinal reattachment, as determined by ophthalmoscopic assessment, fundus photography, and optical coherence tomography (OCT) at the end of the 6-month observation period. All of the photograph-derived outcomes were determined via the independent grading of retinal photographs by scorers at the Fundus Photograph Reading Center which were unaware of the treatment assignments.

The secondary outcomes included visual acuity (VA), intraocular pressure (IOP), intraocular hemorrhage, and degree of recurrent PVR.

Visual acuity was stratified into the following categories: no light perception was scored as 0, light perception was scored as 1, hand motion perception was scored as 2, finger count perception was scored as 3, ≥20/400 was scored as 4, and ≥20/200 was scored as 5 [17]. Vision was considered as improved when VA progressed to the next category; the same when the VA category was unchanged; or worse when VA regressed to a lower category.

Intraocular pressure greater than 25 mmHg was considered to be elevated, and the eye was considered to be hypotensive when IOP was 5 mmHg or less. The PVR grade was defined according to the updated classification of retinal detachment with PVR [18].

The adverse events recorded during this clinical trial were severe ocular inflammation, endophthalmitis, and sympathetic ophthalmia.

### 2.5. Follow-Up

Feasibility was assessed during the surgery. The efficacy and safety variables were assessed at 1 day before surgery (baseline) and at 3 days, 1 week, 2 weeks, 4 weeks, 3 months, and 6 months after surgery.

### 2.6. Statistical Methods

Generalized estimating equations (GEEs) for small samples were used to estimate the changes in VA and IOP from one day before surgery to 6 months postoperatively and at specific time points [19]. All effect estimates, 95% confidence intervals (CIs), and *P* values (*α* = 0.05) were generated using SPSS (Version 16; SPSS Inc., Chicago, IL, USA).

## 3. Results

A total of 9 eyes of 9 consecutive patients were included. The baseline demographic and ocular characteristics are summarized in Table 1. Six adults and three children were recruited. Seven of the nine patients suffered from ocular penetration, and three underwent intraocular surgery twice. Only one patient was phakic; all others were aphakic. The mean duration of silicone oil tamponade was 9.22 ± 14.61 months (range 2–48 months). The mean duration from retinal redetachment to the surgery described herein was 47.78 ± 44.64 days (range 4–150 days).

Fundus characteristics at intraoperative examination are summarized in Table 2. All of the eyes exhibited established PVR (grade C), but the PVR types of these eyes varied. Retinal redetachment was located in the inferior hemifield in all of the patients, and redetachment extended to the macula in two patients. The mean clock length of the detached retina was 5.78 ± 1.64 clock hours (range 4–9 clock hours).

### 3.1. Feasibility Studies

Multiendodiathermy with retinectomies was feasible and efficient. The surgeon completed this modified retinotomy procedure using conventional PPV instruments, and the surgery required a mean 28.00 ± 9.06 minutes from the end of original silicone oil removal to the end of fresh silicone oil application (rang 20–45 minutes).

### 3.2. Efficacy Evaluations

**Figure 2:**
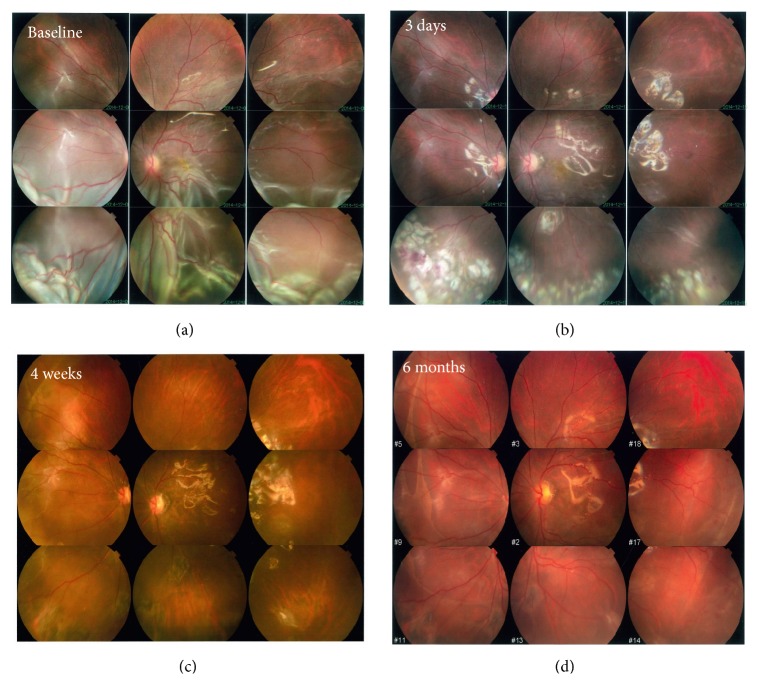
Serial fundus photographs of radial retinotomies with endodiathermy in a chronological order. (a) At baseline, a detached and shortened retina caused by severe PVR was observed. (b) During surgery, a series of retinotomies were created via endodiathermy, and the shortened retina was successfully relaxed. Three days after the operation, the white-marked edges of the retina, indicating the area of endodiathermy and endophotocoagulation, remained detectable. ((c), (d)) The retina was attached at 1 and 6 months after surgery, and no apparent PVR was observed.

Seven of the nine patients exhibited retinal reattachment by the 6-month follow-up assessment (success rate, 78%; 95% CI, 40%–97%). Specifically, all of the adults (100%), but only one of three children (33%), exhibited retinal reattachment.  Figure 2 shows serial photographs of the fundus in chronological order.

**Figure 3:**
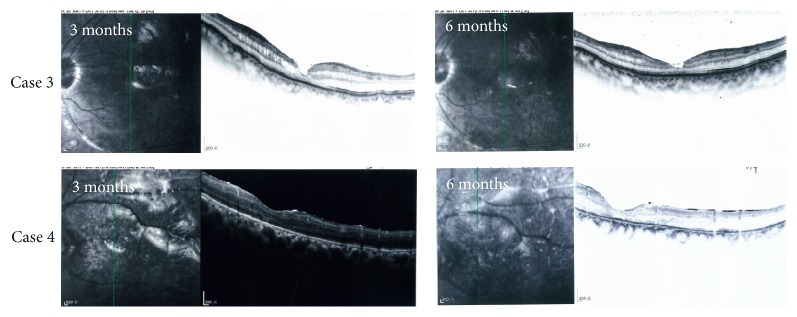
Attachment of the retina as demonstrated by OCT, at 3 and 6 months postoperatively. All the retinal reattachments were assessed relying on indirectly ophthalmoscopy. OCT images were supplied for additionally objective evidences.

All the retinal reattachments were assessed relying on indirectly ophthalmoscopy. Fundus photograph and OCT images were supplied for additionally objective evidences. OCT images showed the retinal attachment at 3 and 6 months postoperatively (Figure 3).

**Figure 4:**
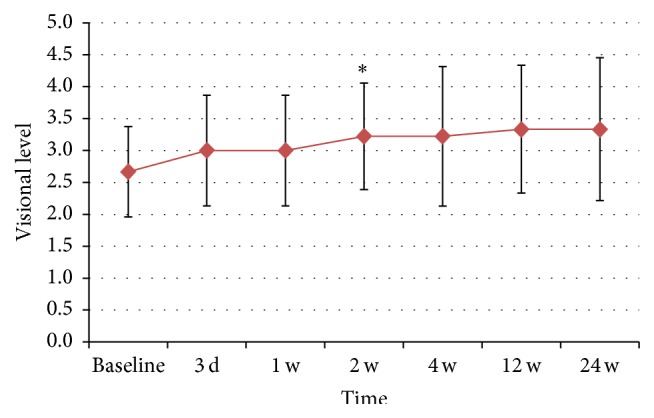
Changes of visual level since baseline to 6 months after operation. The GEE analysis showed that average visual level improved after the operation, peaked at 2 weeks (*P* < 0.05), and kept stable in the following 6 months (*P* > 0.05). Visual acuity (VA) was stratified into the following categories: no light perception was scored as 0, light perception was scored as 1, hand motion perception was scored as 2, finger count perception was scored as 3, ≥20/400 was scored as 4, and ≥20/200 was scored as 5 [17].  ^*∗*^The change of VA was significant from baseline to 2 weeks after the operation.

Visual acuity (VA) improved in 6 patients and was preserved in 1 patient; however, VA was decreased in 2 children (Table 3, Figure 4). GEE analysis showed that the average VA changes were not significant over the 6-month study period (*β* = 0.11; 95% CI, −0.18 to 0.40; *z* = 0.75; *P* = 0.45), but it improved and peaked at 2 weeks after the operation (*β* = 0.20; 95% CI, 0.03 to 0.37; *z* = 2.36; *P* = 0.018) and remained stable for the following 6 months.

### 3.3. Safety Evaluation

**Figure 5:**
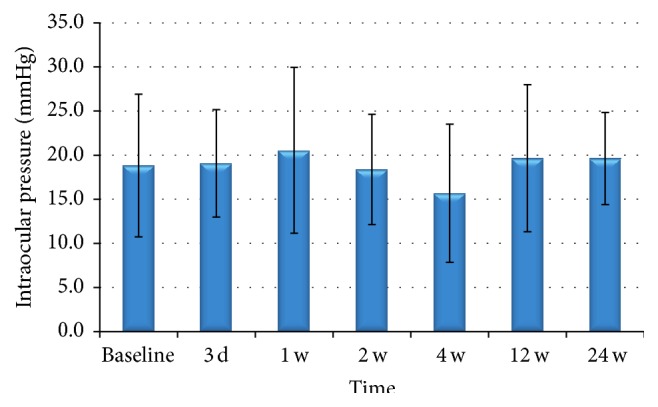
Changes of IOP since baseline to 6 months after operation. The GEE analysis showed that average IOP fluctuated from 15.69 to 19.62 mmHg, and the changes were not significant during the 6-month study (*P* > 0.05).

Slight intraocular hypertension was observed in five patients (55%), but no hypotension was observed (0%) (Table 3, Figure 5). GEE analysis showed that the average IOP fluctuated from 15.69 to 19.62 mmHg; these changes were not significant over the 6-month study period (*β* = −0.006; 95% CI, −0.35 to 0.48; *z* = −0.30; *P* = 0.76).

**Figure 6:**
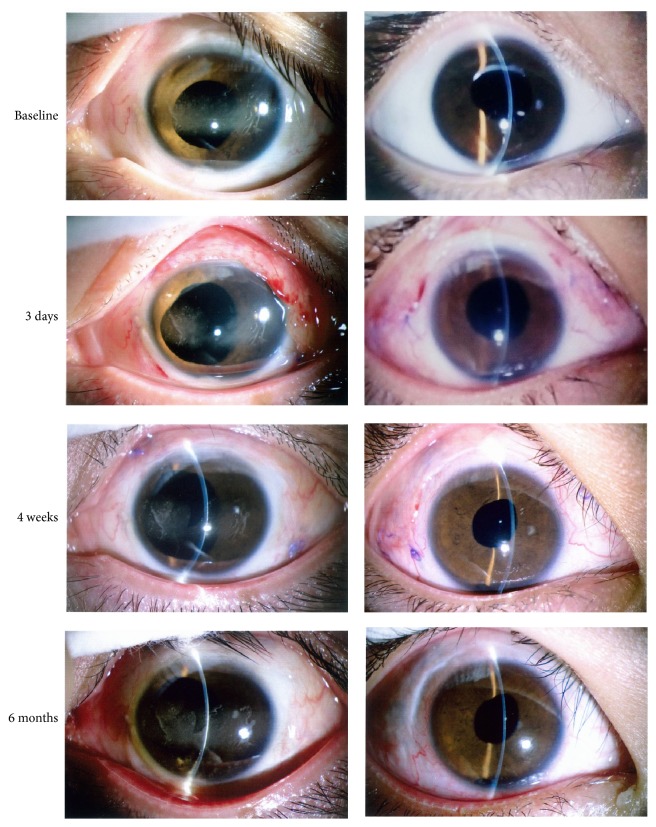
Photographs of ocular anterior segment. Conventional level of inflammation was observed after surgery and resolved in the first 1 or 2 weeks. No obvious inflammation was observed in the cornea or anterior chamber.

No intraocular hemorrhage was observed during or after surgery. Postoperatively, the inflammation of the conjunctiva was similar to that observed following conventional surgery, and the inflammation resolved within 1 or 2 weeks after surgery (Figure 6). No evident inflammation was observed in the cornea or the anterior chamber. No iris neovascularization was observed. No adverse events were observed during the study.

**Figure 7:**
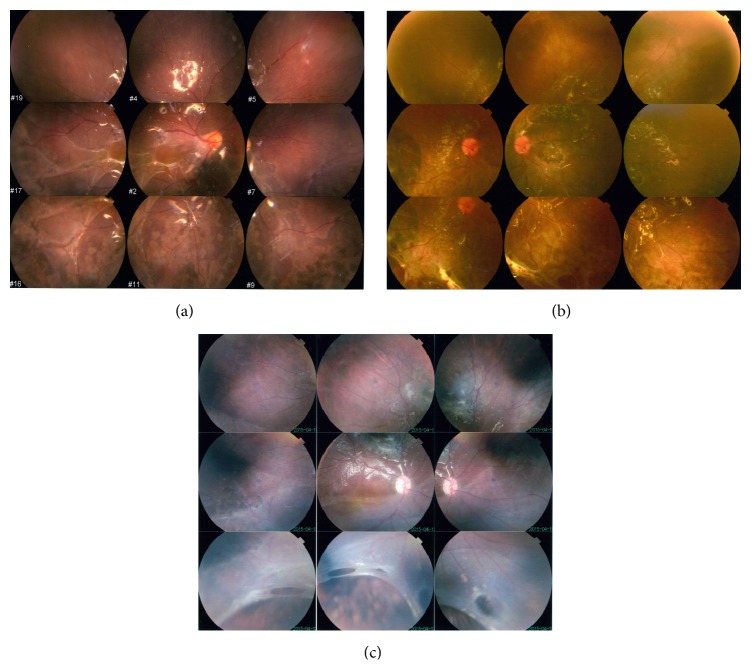
Fundus photographs of three children after surgery. (a) Diffuse proliferation can be observed in one child at 6 months. (b) Diffuse proliferation and peripheral retinal redetachment in 8 o'clock can be observed at 6 months. (c) The edge of the defective retina rolled inwardly to induce the peripheral retinal redetachment since 3 days after surgery.

Diffuse proliferation was observed in the children (Figure 7). The edges of the defective retina rolled inwardly and induced peripheral retinal redetachment. In one child who preoperatively presented with a large, 6-optic disc area defect of the peripheral retina, redetachment was observed as early as 3 days after surgery.

## 4. Discussion

Retinal redetachment of silicone oil-filled eyes is a complicated type of detachment. Relaxing retinectomy was formerly required in these cases, but this procedure may be accompanied with serious complications. The current study proposed a modified technique of radial retinotomies with endodiathermy and preliminarily explored its feasibility, efficacy, and safety for the treatment of severe PVR.

Retinectomy is essential for the relief of extensive retraction, and the reattachment rate associated with this procedure is increasing. In the Silicone Study, a poor anatomic prognosis was observed in 50% of patients undergoing retinectomy [20]. More recent case series have reported better anatomic outcomes, with success rates ranging from 47 to 85% based on heterogeneous collections of retinal detachment cases [6, 12, 15, 21]. The current study examined a homogeneous group of patients with recurrent, inferior retinal detachment of silicone oil-filled eyes that had been traumatized. Thus, results indicate that this modified surgery can lead to favorable outcomes in adults with severe PVR when conventional methods have failed.

However, the retinas of two children (a 7- and a 12-year-old) failed to reattach. Fundus photographs revealed that postoperative PVR was significantly more severe in the pediatric eyes. Importantly, one child with a 6-optic disc area defect of the peripheral retina exhibited redetachment as early as 3 days after surgery, although the surgeon cut the inward curling edge of his retina during the surgery. These results indicate that this modified surgery might have low success rates specifically among children with large peripheral retinal defects.

The VA results of retinectomy in previous case series have varied markedly, with VA improving in 20–89% of patients but deteriorating in 13–41% of patients [20, 21]. In a mixed series of 41 eyes, 63% of which were traumatized, Kolomeyer et al. reported that VA deteriorated in 22% of cases [16]. Lewis and Iverson identified larger retinectomies as a risk factor for poorer final visual function [22, 23]. In the current study, stabilization or improvement of VA was observed in 78% of the patients. This result might be attributed to the reduced number of cuts, which were limited to the shortened retina, and might indicate that this novel surgery remains worthwhile.

Retinectomy has been advocated to treat intractable glaucoma because the absorption of intraocular fluid from the large area of the exposed RPE can reduce IOP [24, 25]. In the Silicone Study, hypotony occurred approximately twice as frequently in eyes undergoing retinectomy than in those that did not (35% versus 17%) [20]. Recent studies reported hypotony rates in retinectomy case series ranging from 11% to 43% [12–14]. The current study did not observe any cases exhibiting hypotony, and 5 patients displayed slight intraocular hypertension. This successful maintenance of IOP might be attributable to the modified cutting approach. The preserved retina between the incisions helped to cover the underlying RPE, potentially reducing the absorption of intraocular fluid.

Intraoperative hemorrhage occurs in retinectomy when extending the cut into the normal retina. The blades of the scissors are typically tangentially directed into the subretinal space to lift the retina away from the RPE. Kolomeyer et al. reported intraoperative hemorrhage in 32% of eyes undergoing retinotomy [16]. However, no intraoperative hemorrhage was observed in the current study. Using this modified procedure, the retina was cut via diathermy with simultaneous hemostasis during perforation. More importantly, the cut was limited to the detached retina, avoiding the dangerous separation of the normal retina from the choroid.

This modified retinotomy is based on meticulous dissection of vitreous base, preretinal and subretinal membranes. To inferior recurrent retinal detachment induced by vitreous base fibrosis alone, this approach is unnecessary and will cause multiple iatrogenic retinal tears. It should be noted that we did not carry out random clinical trials to compare the difference between relaxing retinectomy and this modified retinotomy. Traditional relaxing retinectomy may also attach the retina well in these patients. Besides, side effect as postsurgery hypotony is avoidable in relaxing retinectomy if the retinectomy is large circumferentially but not anteroposteriorly. One other important limitation of this procedure was that all the eyes continued to be filled with silicone oil. Eyes that require retinotomy are usually accompanied with severe PVR and extensive atrophy of retina. In retrospective studies recruiting 56 and 145 patients for retinectomies, silicone oil is still the tamponade agent in the eyes of 50% patients [14, 26]. We attempted to remove the silicone oil in patients whose retina appears to be well attached. However, considering the severe ocular trauma and retinal atrophy, we have to maintain the silicone oil tamponade in place to guarantee a surgical benefit to the patients.

## 5. Conclusions

In conclusion, radial retinotomies with endodiathermy was shown to be feasible, effective, and safe in selected cases of inferior contracted retina without vitreous base fibrosis over a 6-month observation period.

## Figures and Tables

**Table 1:** Demographic and ocular characteristics at baseline examination.

Patient number	Sex	Age (years)	Type of ocular trauma	Number of intraocular surgeries	Lens state	Number of silicone oil tamponades	Duration of silicone oil tamponade (months)	Duration of retinal redetachment (days)
1	M	21	Penetration	1	Aphakic	1	5	45
2	M	23	Penetration	2	Aphakic	1	3	30
3	M	28	Penetration	1	Aphakic	1	6	60
4	M	30	Penetration	1	Aphakic	1	5	150
5	M	50	Penetration	1	Aphakic	1	6	60
6	F	39	Contusion	1	Phakic	1	2	7
7	M	7	Penetration	2	Aphakic	1	5	60
8	M	8	Penetration	2	Aphakic	1	3	14
9	M	12	Contusion	1	Aphakic	1	48	4

M: male; F: female.

**Table 2:** Fundus characteristics at intraoperative examination.

Patient	PVR classification	PVR type	Number of retinal holes	Existence of retinal defect	Total area of retinal defect (optic disc are)	Clock hours of retinal detachment	Detachment of macula
1	Grade C P1	Type 1	1	No	0	7	No
2	Grade C P1	Type 1	3	Yes	3	5	Yes
3	Grade C A4	Type 4	1	Yes	10	9	No
4	Grade C A6	Type 1	1	No	0	7	Yes
5	Grade C A1	Type 5	1	Yes	4	5	No
6	Grade C P1	Type 5	0	No	0	4	No
7	Grade C A2	Type 2	1	No	0	4	No
8	Grade C P3	Type 2	0	No	0	5	No
9	Grade C A2	Type 4	2	Yes	6	6	No

The PVR grade was defined according to the updated classification of retinal detachment with PVR [18].

**Table 3:** The changes of visual level and IOP during the study.

Patients	VA (Snellen scale)		IOP (mmHg)	
	Baseline	6 months	Baseline	6 months
Case 1	20/2000	20/100	12.3	23.0
Case 2	CF/10 cm	FC/10 cm	7.5	15.5
Case 3	HM/20 cm	20/2000	18.4	18.1
Case 4	HM/10 cm	HM/40 cm	12.4	16.3
Case 5	HM/20 cm	FC/5 cm	28.0	18.5
Case 6	20/2000	20/200	20.0	21.5
Case 7	HM/40 cm	FC/5 cm	23.4	21.0
Case 8	20/400	20/400	14.8	12.3
Case 9	20/1000	HM/10 cm	32.6	30.4

VA: visual acuity; HM: hand motion; CF: counting fingers; IOP: intraocular pressure. The visual acuities were obtained with Snellen charts.
